# Supplementary material for: Community-associated quinolone-resistant and extended-spectrum beta-lactamase-producing Escherichia coli isolates are similar to clinical infection isolates by sequence type and resistome
Source: mSystems. 2026 Jan 12;11(2):e01591-25. doi: 10.1128/msystems.01591-25 (PMC12911353; doi:10.1128/msystems.01591-25)
Supplement: Fig. S1 — SNP analysis histograms. [file msystems.01591-25-s0001.pdf]

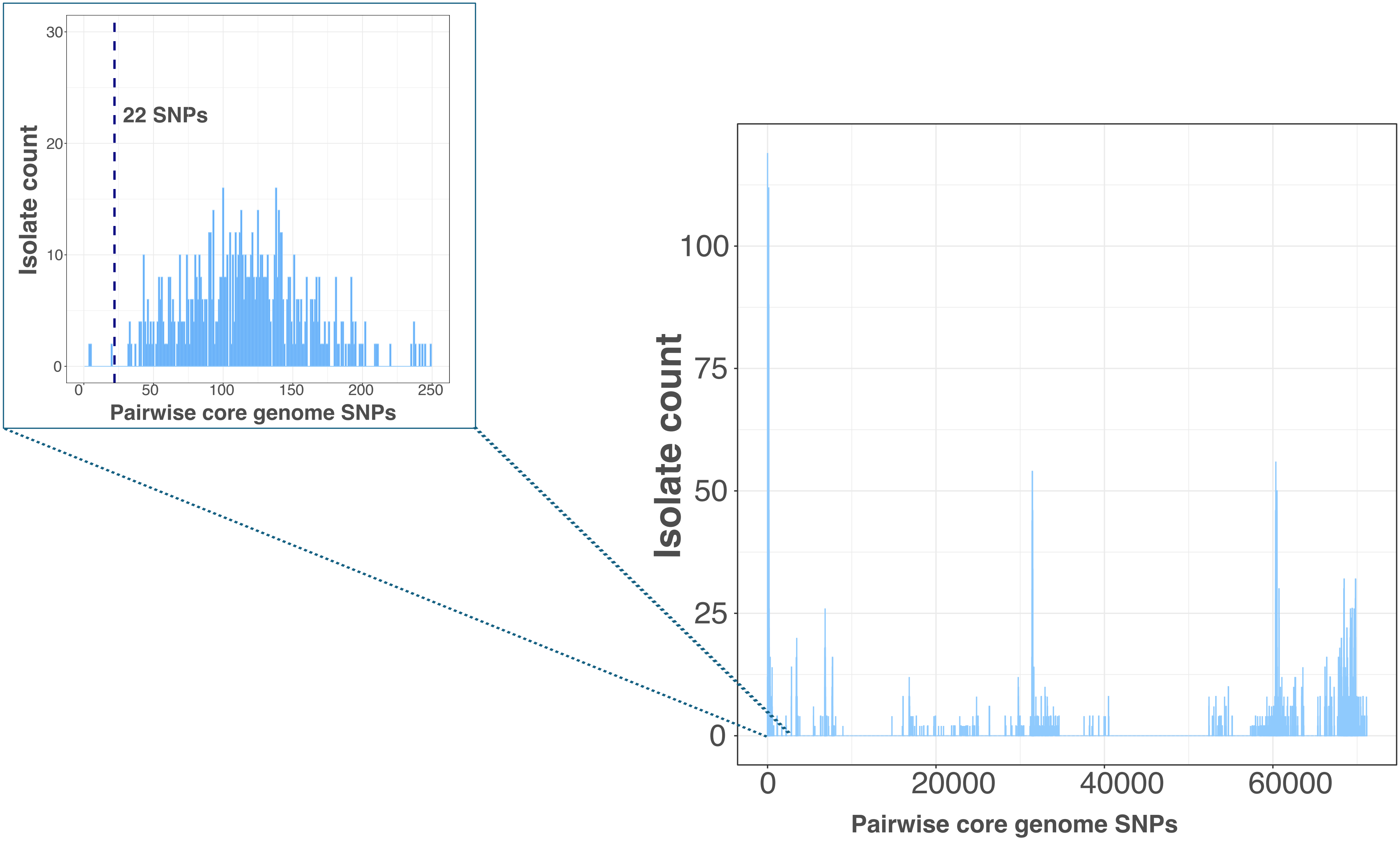

Supplemental Figure 1: Pairwise analysis to determine empirical SNP cutoff. Pairwise core-genome SNPs between all 75 CA *E. coli* isolates of this study. SNP = single-nucleotide polymorphism.
