# Supplementary material for: Community-associated quinolone-resistant and extended-spectrum beta-lactamase-producing Escherichia coli isolates are similar to clinical infection isolates by sequence type and resistome
Source: mSystems. 2026 Jan 12;11(2):e01591-25. doi: 10.1128/msystems.01591-25 (PMC12911353; doi:10.1128/msystems.01591-25)
Supplement: Fig. S2 — ARG annotations of within-patient co-colonizing isolates. [file msystems.01591-25-s0002.pdf]

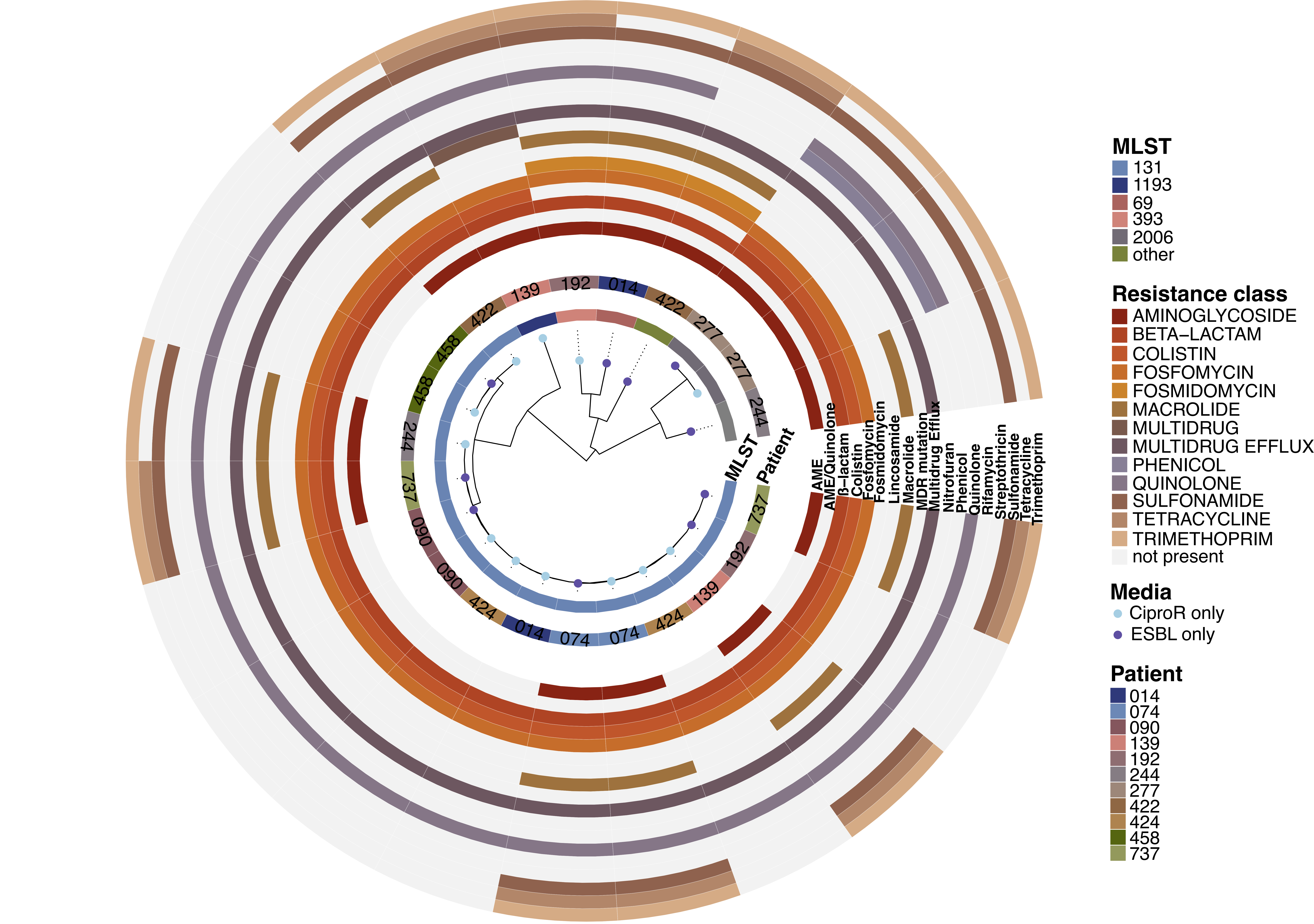

Supplemental Figure 2: Antimicrobial resistance features of 22 isolates showing within-patient co-colinization. Maximum-likelihood core genome phylogenetic tree of 22 intra-patient isolate pairs separated by more than 22 core-genome SNPs annotated by media, MLST, patient, and resistance elements annotated by AMRFinderPlus, displayed by class. SNPs = single-nucleotide polymorphisms; MLST = multi-locus sequence type; ESBL = extended-spectrum beta-lactamase; CiproR = ciprofloxacin resistant.
