# Supplementary material for: Community-associated quinolone-resistant and extended-spectrum beta-lactamase-producing Escherichia coli isolates are similar to clinical infection isolates by sequence type and resistome
Source: mSystems. 2026 Jan 12;11(2):e01591-25. doi: 10.1128/msystems.01591-25 (PMC12911353; doi:10.1128/msystems.01591-25)
Supplement: Fig. S3 — ARG and AST information for all isolates in the present study. [file msystems.01591-25-s0003.pdf]

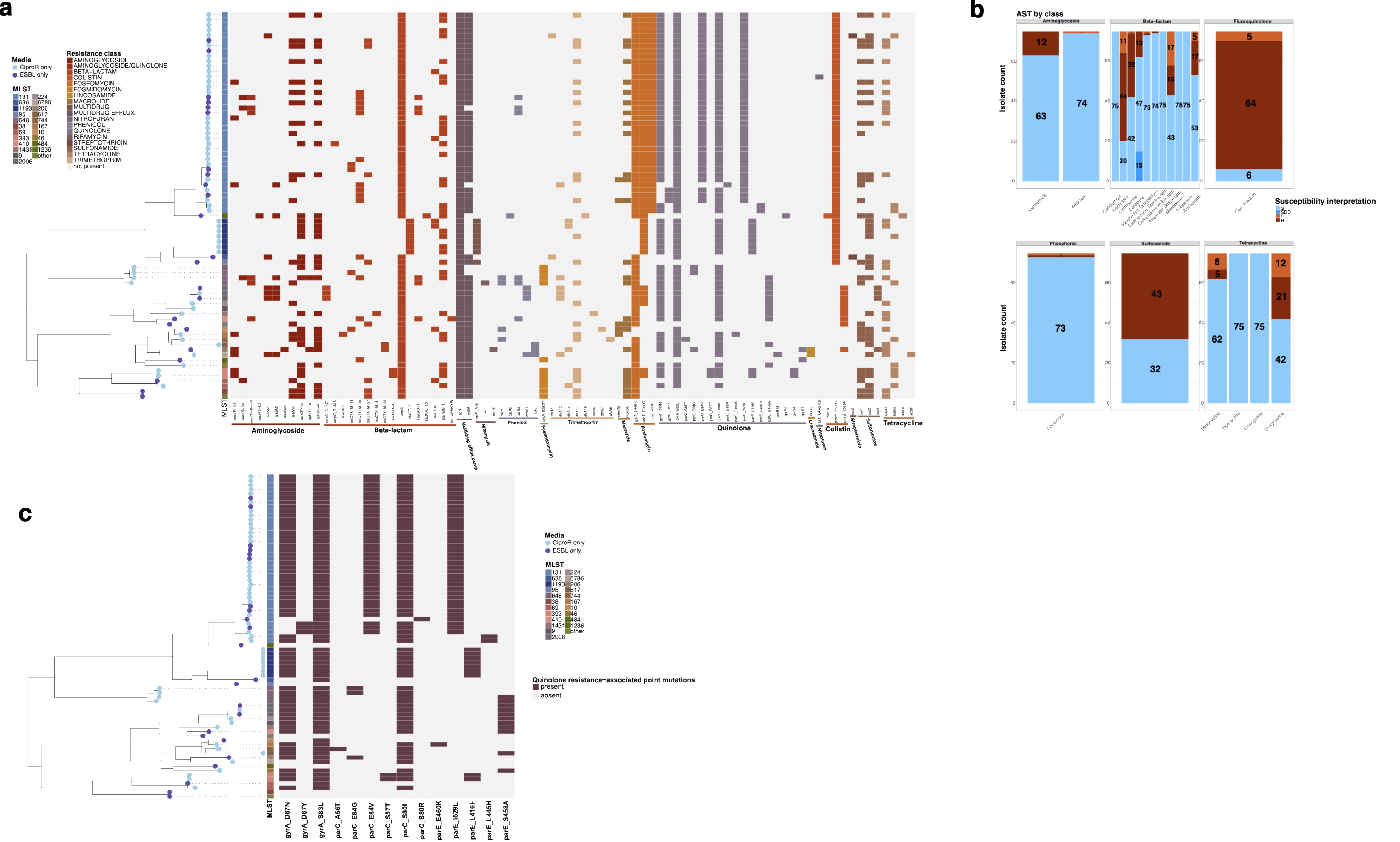

Supplemental Figure 3: All antimicrobial resistance genes and antimicrobial susceptabilities for 75 CA isolates. a) Maximum-likelihood core genome phylogenetic tree annotated with media, MLST, and resistance genes and mutations. Resistance elements are groupes and colored by class. b) Stacked barplots of susceptibility interpretations for all tested antimicrobials, grouped by class. Numbers within bars indicate the number of isolates in that group. c) Maximum-likelihood core genome phylogenetic tree annotated with media, MLST, and specific point mutations associated with quinolone resistance. S = susceptible; SDD = susceptible dose-dependent; I = intermediate; R = resistant; ESBL = extended-spectrum beta-lactamse; CiproR = ciprofloxacin resistant.
