# Supplementary material for: Community-associated quinolone-resistant and extended-spectrum beta-lactamase-producing Escherichia coli isolates are similar to clinical infection isolates by sequence type and resistome
Source: mSystems. 2026 Jan 12;11(2):e01591-25. doi: 10.1128/msystems.01591-25 (PMC12911353; doi:10.1128/msystems.01591-25)
Supplement: Fig. S4 — ARG annotations of cross-patient similar isolates. [file msystems.01591-25-s0004.pdf]

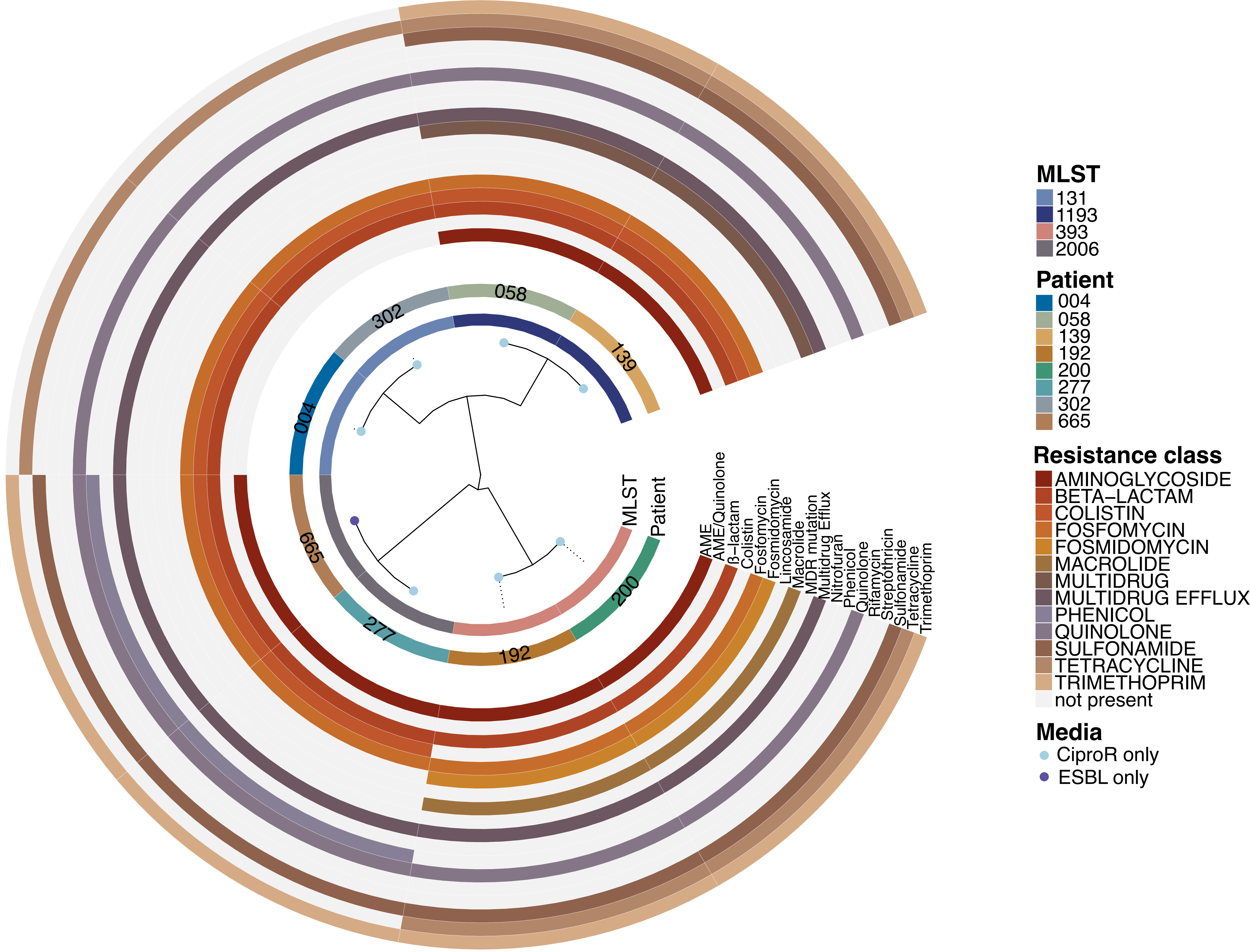

Supplemental Figure 4: Antimicrobial resistance features of eight inter-patient similar isolates. Maximum-likelihood core genome phylogenetic tree of eight inter-patient isolate pairs within 22 core-genome SNPs annotated by media, MLST, patient, and resistance elements as annotated by AMRFinderPlus, displated by class. SNPs = single-nucleotide polymorphisms; MLST = multi-locus sequence type; ESBL = extended-spectrum beta-lactamase; CiproR = ciprofloxacin resistant.
