# Supplementary material for: Community-associated quinolone-resistant and extended-spectrum beta-lactamase-producing Escherichia coli isolates are similar to clinical infection isolates by sequence type and resistome
Source: mSystems. 2026 Jan 12;11(2):e01591-25. doi: 10.1128/msystems.01591-25 (PMC12911353; doi:10.1128/msystems.01591-25)
Supplement: Fig. S5 — ST131 accessory genome information. [file msystems.01591-25-s0005.pdf]

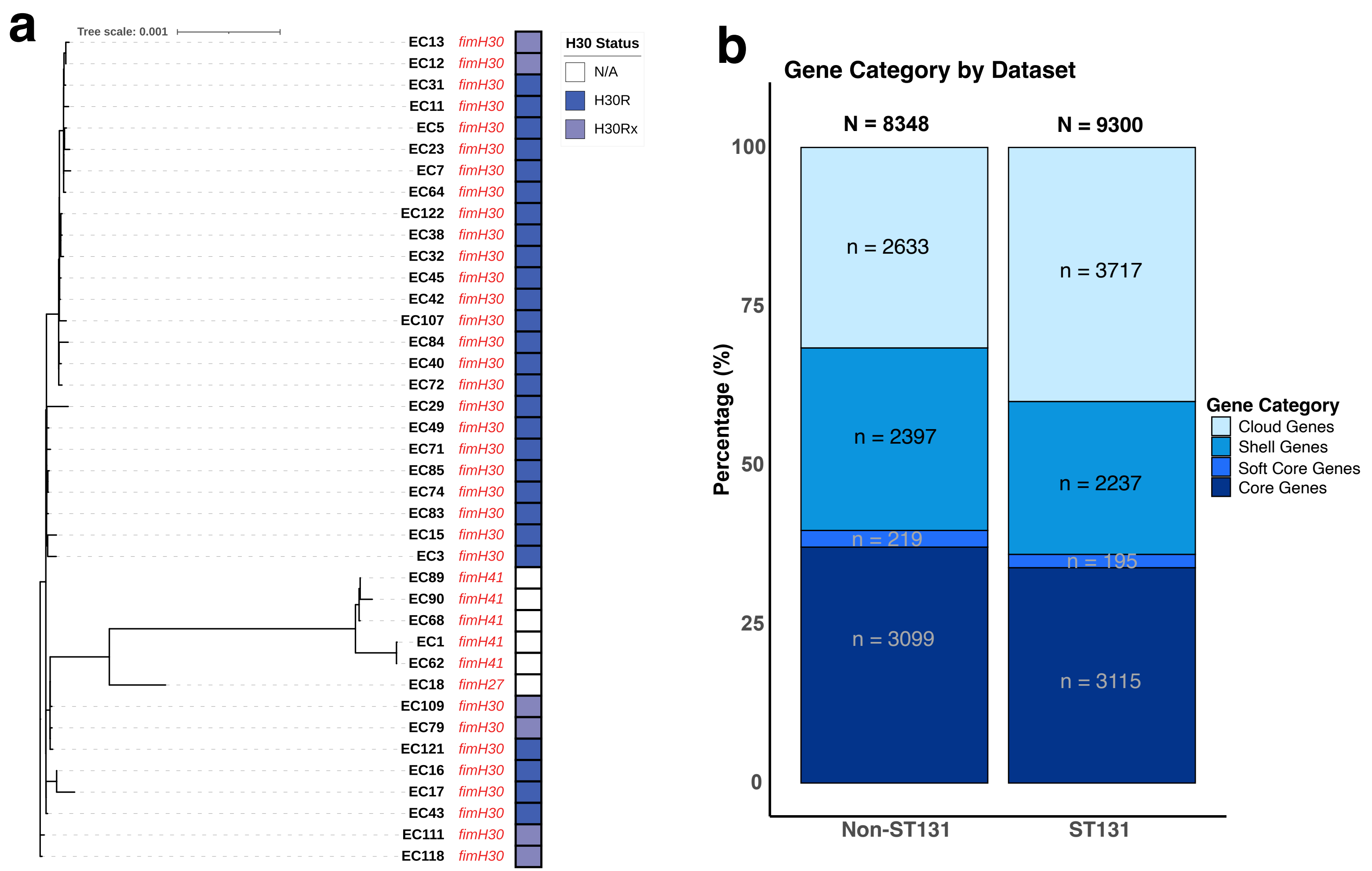

Supplemental Figure 5: ST131 phylogeny and accessory gene content. a) Maximum-likelihood core genome phylogenetic tree annotated with FimH allele and H30 status. b) Core and accessory gene content for ST131 and nonST131 genomes.
ST = sequence type.
