# Supplementary material for: Community-associated quinolone-resistant and extended-spectrum beta-lactamase-producing Escherichia coli isolates are similar to clinical infection isolates by sequence type and resistome
Source: mSystems. 2026 Jan 12;11(2):e01591-25. doi: 10.1128/msystems.01591-25 (PMC12911353; doi:10.1128/msystems.01591-25)
Supplement: Fig. S6 — Network comparison of the Mahmud et al. comparison isolates with the present study isolates. [file msystems.01591-25-s0006.pdf]

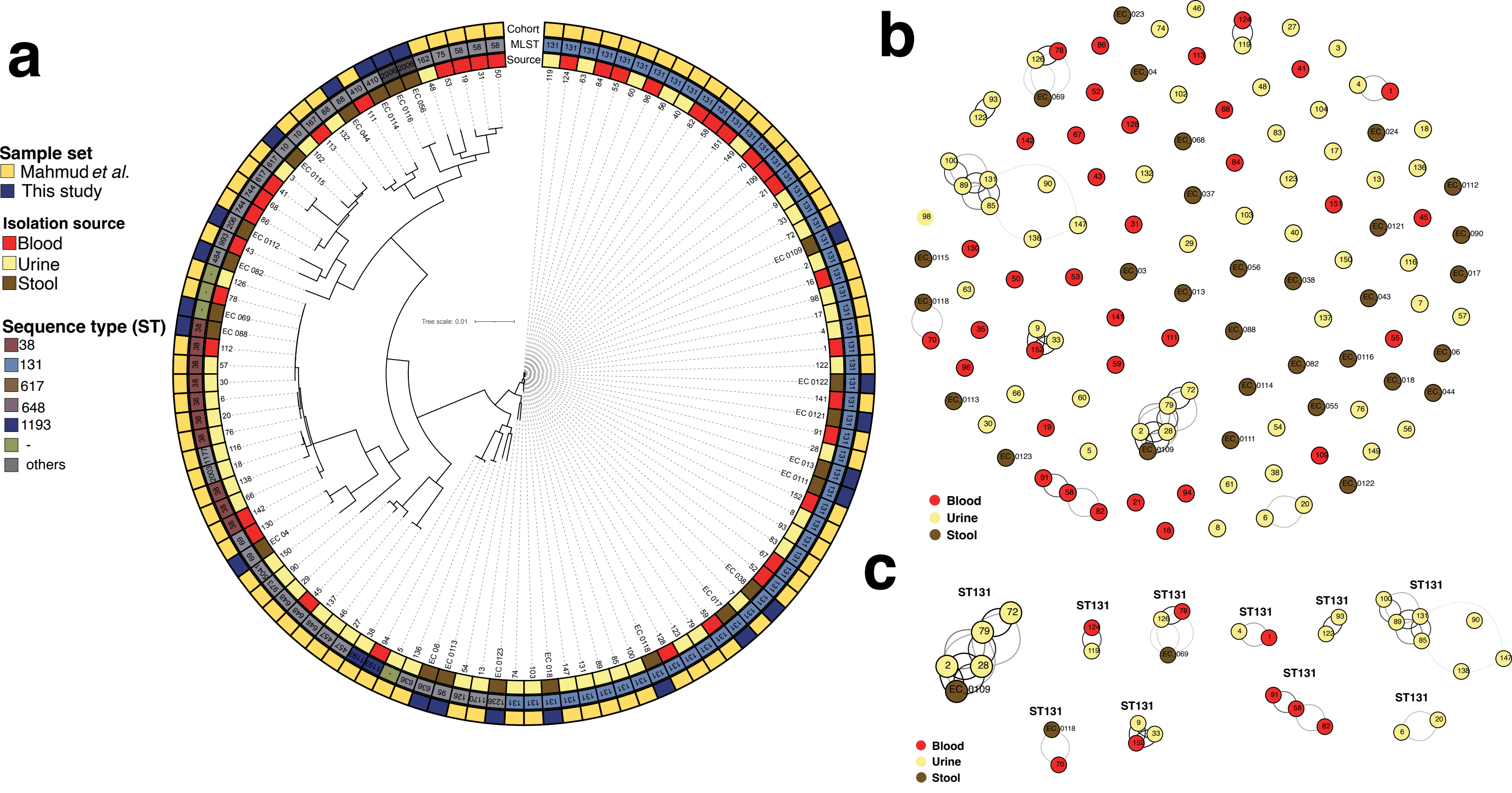

Supplemental Figure 6: Continued surveillance and network analysis by isolate source. a) Maximum-likelihood core genome phylogenetic tree annotated with the respective isolate set, isolate source, and reported ST. b) Network analysis of CA ESBL *E. coli* in this study and the ESBL *E. coli* clinical isolates from Mahmud *et al.* colored by isolate source. c) A subset of the network in b. ST = sequence type.
