# Supplementary material for: Community-associated quinolone-resistant and extended-spectrum beta-lactamase-producing Escherichia coli isolates are similar to clinical infection isolates by sequence type and resistome
Source: mSystems. 2026 Jan 12;11(2):e01591-25. doi: 10.1128/msystems.01591-25 (PMC12911353; doi:10.1128/msystems.01591-25)
Supplement: Table S1 — Present patient and Mahmud et al. patient descriptions. [file msystems.01591-25-s0007.docx]

**Supplemental Table 1:** Patient characteristics from CA ESBL *E. coli* in this study and in Mahmud *et al*.

| **Variable** | **This study**  **N=64**  **N (%)** **or median (range)** | **Mahmud *et al*.**  **N=87**  **N (%) or median (range)** |
| --- | --- | --- |
| **Specimen type** |  |  |
| Stool | 64 (100) | NA |
| Blood | NA | 34 (39) |
| Urine | NA | 53 (61) |
| Age | 62 (18 – 94) | 62 (23 – 93) |
| Female | 31 (48) | 49 (56) |
| Non-white or unknown race | 22 (34) | 44 (51) |
| **Patient status at time of specimen collection** |  |  |
| Inpatient / observation | 44 (69) | Unknown |
| Outpatient | 17 (26) | Unknown |
| ED | 3 (5) | Unknown |
| **Medications in 12 weeks prior to specimen collection** |  |  |
| Any antibiotics | 32 (50) | 67 (77) |
| Penicillins | 8 (13) | 14 (16) |
| Cephalosporins | 11 (17) | 45 (52) |
| Carbapenems | 0 (0) | 14 (16) |
| Quinolones | 14 (22) | 23 (26) |
| Immunosuppressant | 15 (23) | 26 (30) |
| Chemotherapy | 12 (19) | 23 (26) |
| **Infection in 12 weeks prior to specimen collection** |  |  |
| Any infection | 14 (22) | Unknown |
| Blood stream infection | 1 (2) | Unknown |
| Gastrointestinal tract infection | 3 (5) | Unknown |
| Pneumonia | 1 (2) | Unknown |
| Soft tissue infection | 2 (3) | Unknown |
| Upper respiratory tract infection | 3 (5) | Unknown |
| Urinary tract infection | 3 (5) | Unknown |
| Other/Unknown infection type | 1 (2) | Unknown |
| **Patient comorbidities** |  |  |
| AIDS | 1 (2) | Unknown |
| Catheter at time of specimen collection | 9 (14) | Unknown |
| Cerebrovascular accident | 6 (9) | Unknown |
| Chronic kidney disease | 12 (19) | Unknown |
| Chronic obstructive pulmonary disease | 4 (6) | Unknown |
| Congestive heart failure | 4 (6) | Unknown |
| Connective tissue disease | 5 (8) | Unknown |
| Dementia | 2 (3) | Unknown |
| Diabetes | 13 (20) | Unknown |
| Inflammatory bowel disease | 13 (20) | Unknown |
| Interstitial cystitis | 2 (3) | Unknown |
| Irritable bowel syndrome | 1 (2) | Unknown |
| Kidney stones | 1 (2) | Unknown |
| Leukemia | 3 (5) | Unknown |
| Liver disease | 4 (6) | Unknown |
| Lymphoma | 2 (3) | Unknown |
| Menopause or post-menopausal | 23 (36) | Unknown |
| Other genitourinary disease | 9 (14) | Unknown |
| Paraplegia or hemiplegia | 5 (8) | Unknown |
| Pelvic floor dysfunction | 1 (2) | Unknown |
| Peptic ulcer disease | 1 (2) | Unknown |
| Peripheral vascular disease | 3 (5) | Unknown |
| Previous bone marrow transplant | 1 (2) | Unknown |
| Previous colectomy | 10 (16) | Unknown |
| Previous solid organ transplant | 8 (13) | Unknown |
| Prostate disease | 9 (14) | Unknown |
| Solid tumor | 22 (34) | Unknown |
| Urinary incontinence | 2 (3) | Unknown |
| Urinary retention | 5 (8) | Unknown |
| Urinary system disease | 12 (19) | Unknown |
